# Effects of Decabromodiphenyl Ether (BDE209) Exposure on Toxicity and Oxidative Stress of Beas-2B Cells

**DOI:** 10.3390/toxics13110987

**Published:** 2025-11-16

**Authors:** Yanan Zhang, Ziyu Xiao, Pu Mao, Fengrui Yang, Yingdi Ma, Bensen Xian, Mingming Fu, Guiying Li

**Affiliations:** 1 Guangdong Basic Research Center of Excellence for Ecological Security and Green Development, Key Laboratory of City Cluster Environmental Safety and Green Development of the Ministry of Education, School of Environmental Science and Engineering, Guangdong University of Technology, Guangzhou 510006, China; 2 Guangxi Key Laboratory of Environmental Pollution Control Theory and Technology, Guilin University of Technology, Guilin 541006, China; 3 State Key Laboratory of Respiratory Disease, Guangzhou Medical University, Guangzhou 510182, China; 4 University Engineering Research Center of Watershed Protection and Green Development, Guilin University of Technology, Guilin 541006, China

**Keywords:** decabromodiphenyl ether (BDE209), Beas-2B, N-acetylcysteine (NAC), oxidative stress, toxicity

## Abstract

Decabromodiphenyl ether (BDE209) has been widely used because of its excellent flame-retardant properties and ability. On the one hand, many studies have shown that the presence of BDE209 can potentially threaten human health and the environment. The production and processing of products containing BDE209 is prohibited except for special applications in China. On the other hand, the study of BDE209 on respiratory cells is not yet fully understood. Consequently, this study aims to investigate the mechanisms of toxic damage and oxidative stress induced by BDE209 exposure in lung epithelial Beas-2B cells. The proliferation of Beas-2B cells under BDE209 exposure was first analyzed by using a real-time label-free cell analyzer (RTCA). Then the cells’ morphological changes were observed using laser confocal microscopy. Subsequently, the effects of BDE209 exposure alone, combined exposure to N-acetylcysteine (NAC) and BDE209, on reactive oxygen species (ROS) levels and antioxidant defense-related factors in Beas-2B cells were analyzed separately. The results show that BDE209 exposure induces the proliferation of Beas-2B cells with a dose-dependent increase in inhibition. Microscopic observation of Beas-2B cells reveals significant damage and death. The levels of ROS are significantly increased (*p* < 0.01), the contents of superoxide dismutase (SOD) and malondialdehyde (MDA) are increased, the contents of catalase (CAT) are decreased, and the activities of glutathione peroxidase (GPX) are first decreased and then increased. However, under the co-exposure of NAC and BDE209, ROS levels are significantly reduced (*p* < 0.01), MDA contents decrease, and SOD activities increase. In summary, BDE209 exposure leads to inhibition of Beas-2B cell proliferation, cellular morphology damage, increased ROS levels, and disturbances in antioxidant defense-related factors. The cells showed toxic damage and oxidative stress. In contrast, NAC can suppress ROS levels, enhance SOD activity, and inhibit GPX activity, thereby alleviating BDE209-induced cellular damage.

## 1. Introduction

Decabromodiphenyl ether (C_12_Br_10_O, CAS Registry No. 1163-19-5, BDE209) is a white or yellowish powder, the most abundant bromide congener of polybrominated diphenyl ethers (PBDEs), and is commonly used as a non-reactive additive flame retardant in building materials, electronics, plastics, textiles, and other materials. BDE209 is one of the most widespread persistent organic pollutants (POPs) [1]. BDE209 has been detected in our atmosphere, soil, water, sediments, and even indoor air and dust, and it is also unavoidable in plants and animals [2,3]. BDE209 is persistent, lipophilic, refractory, and easily able to bioconcentrate, which seriously endangers plant, animal, and human health. Since the discovery of its hazards, studies have expanded significantly, especially in the context of the respiratory system, covering areas such as toxicology, co-toxicity, and contaminant degradation [4]. In addition to the respiratory system, BDE209 has been demonstrated to induce cytotoxic effects in various other cell types. For instance, studies have shown that BDE209 exposure leads to oxidative stress and apoptosis in hepatocytes [5]. Similarly, it has been reported to cause impairments in neuronal development and function [6]. These findings across different cell models underscore the broad toxic potential of BDE209 and justify the need to further elucidate its specific effects and mechanisms on human airway epithelium.

Normal intracellular homeostasis is associated with a dynamic balance between the production of ROS radicals, including superoxide anion radical (O^2−^), hydroxyl radical (·OH), hydrogen peroxide radical (HO_2_), hydrogen peroxide (H_2_O_2_), etc. H_2_O_2_ can penetrate most cell membranes, and when it crosses the cell, it reacts with intracellular iron to produce ·OH. It was related to the dynamic balance between manufacture and the antioxidant defense system, which the body produces naturally but whose levels may be elevated by environmental influences such as UV radiation, pollution, and stress [7]. High levels of ROS trigger a free radical chain reaction in which the antioxidant system is activated to scavenge excess free radicals. The antioxidant system consists of two types: an enzymatic antioxidant system including glutathione peroxidase (GPX), superoxide dismutase (SOD), catalase (CAT), and glutathione reductase (GR), and a non-enzymatic antioxidant system including vitamin C, GSH, and the trace elements copper (Cu) and iron (Fe). When the number of free radicals exceeds the level of the body’s antioxidant defenses, resulting in a dysregulation of redox homeostasis, oxidative stress is induced and intracellular biomolecules (lipids, etc.) are destroyed, causing cellular damage [8].

This process is accompanied by the production and accumulation of several biomarkers, including ROS, antioxidant enzymes, MDA, and others [9]. Oxidative stress has been linked to the development of many diseases and pathologies, such as diabetes and its complications, bone diseases, neurological disorders, etc., and can even lead to cancer [10,11,12,13].

N-acetylcysteine (NAC) is an antioxidant that acts as a cysteine bio-acetylation precursor and can increase the synthesis of reduced glutathione (GSH) and also acts as a scavenger to directly scavenge reactive oxygen species (ROS) [14]. It attenuates oxidative stress damage induced by a variety of pollutants and prevents apoptosis through multiple pathways [15,16].

Beas-2B cells are infectious, non-malignant, normal lung cells derived from the epithelium of the human bronchi. They are used as a model for lung epithelial cell lines in many in vitro studies related to toxicology, drug screening, etc. [17]. Liu has demonstrated the medicinal value of a Pinus sylvestris that increased the cell viability of Beas-2B cells exposed to lipopolysaccharide (LPS) [18]. AHR and PPAR-FATP1 signal transduction are potential therapeutic targets for intervening in benzo(a)pyrene (Bap)-induced toxicity and related disorders by investigating benzo(a)pyrene (Bap)-exposure-induced Beas-2B cells [19].

To date, few in vitro experiments have explored the effects of BDE209 on Beas-2B cells alone, and to fill this gap in the literature, we designed the present study to examine the effects of BDE209 on Beas-2B cells in terms of cytotoxicity, morphology, and oxidative stress. The effect of NAC addition on BDE209-induced oxidative stress in Beas-2B cells was also investigated for subsequent programmed cellular necrosis and lung-related diseases.

## 2. Materials and Methods

### 2.1. Chemicals

BDE209 (99% pure) was purchased from J&K Scientific (Beijing, China). Dulbecco’s Modified Eagle Medium (DEME-F12), Fetal Bovine Serum (FBS), phosphate-buffered saline (PBS), penicillin–streptomycin and trypsin-EDTA were obtained from Gibco (Thermo Fisher Scientific, Waltham, MA, USA). Dimethyl Sulfoxide (DMSO) was obtained from Aladdin (Shanghai, China).

The complete medium was prepared with DMEM-F12, 5% FBS, and 1% penicillin–streptomycin solution (final concentrations: 100 U/mL penicillin and 100 µg/mL streptomycin).

### 2.2. Cell Culture

The Beas-2B cell line was a generous gift from the State Key Laboratory of Respiratory Diseases, Guangzhou Medical University (Guangzhou, China). Cells were cultured in Dulbecco’s Modified Eagle Medium (DEME-F12) supplemented with 10% FBS and 1% penicillin–streptomycin and maintained at 37 °C in a humidified atmosphere of 5% CO_2_. All experiments were conducted using cells within a narrow passage range (P5–P15) to guarantee genetic and functional consistency and to avoid artifacts associated with long-term culture. After adherent growth to 80% and washing twice with PBS, cells were digested by using trypsin containing 0.25% EDTA. Add complete medium to terminate the process, centrifugation, and resuspension to obtain cell suspension.

### 2.3. Determining IC_50_ Values Using RTCA

Real-Time Cellular Analysis (RTCA, Aceabio, Hangzhou, China) technology can track cell morphology, adhesion proliferation, and differentiation in real-time, dynamically and quantitatively, making it a more intuitive and convenient assay for cytotoxicity detection.

The half-maximal inhibitory concentration (IC_50_) of BDE209 was first determined [20]. The selected concentration range (1–100 µM, specifically 1, 5, 10, 20, 40, 80, and 100 µM) was designed to include concentrations expected to cause minimal cytotoxicity as well as those leading to near-complete cell apoptosis.

Briefly, background impedance was measured after adding culture medium to an E-plate. A Beas-2B cell suspension was then seeded into the E-plate at a density of 1 × 10^4^ cells/well, as optimized in preliminary experiments. The plate was left undisturbed for 30 min at room temperature in a biosafety cabinet to allow uniform cell distribution before being transferred to the RTCA station inside a cell culture incubator. After 24 h of attachment, when the Cell Index reached approximately 1.2 (indicating the logarithmic growth phase), the cells were treated with the respective BDE209 concentrations (1, 5, 10, 20, 40, 80, and 100 µM) or with 0.5% DMSO as a vehicle control. Cellular proliferation and cytotoxicity were monitored in real time, the cell proliferation curves were plotted (Figure S1). The calculated IC_50_ values of BDE-209 for Beas-2B cells at 6, 12, 24, and 48 h were 30.9, 43.0, 74.5, and 79 µM, respectively, as shown in Figure S2 and Table S1.

Based on these results, an exposure duration of 24 h was selected for subsequent experiments. The corresponding 24 h IC_50_ value (74.5 µM) was used to determine the final BDE209 test concentrations for the main study: 5, 20, 35, 50, and 65 µM.

### 2.4. Cytotoxicity Exposure

BDE-209 Exposure of Beas-2B Cells: Cells were seeded in 6-well plates at a density of 3 × 10^5^ cells per well and cultured in 3 mL of complete medium until they reached approximately 80% confluence. The medium was then replaced with 3 mL of serum-free medium for a 4 h starvation period. Following starvation, the cells were exposed by replacing the medium with 3 mL of complete medium containing 0.1% FBS and the respective concentrations of BDE-209 stock solution (15 µL per well, yielding final BDE-209 concentrations of 5, 20, 35, 50, and 65 µM). The final concentration of DMSO (the solvent for BDE-209) was maintained at 0.5% in all treatment groups. After 24 h of exposure, the culture supernatant was collected, and enzyme activities were measured using a microplate reader (Thermo Scientific, Waltham, MA, USA). The remaining cells were used for the detection of intracellular ROS.

We measured the potential toxic effects of 0.5% DMSO and compared them with the negative control to eliminate erroneous results. Because of the high photo-degradability of BDE209, all analyses were performed under dark conditions. We set the cell culture supplemented with 0.5% DMSO as the control group. For the BDE209 treatment groups, we established a concentration range of 0–65 μM BDE209 diluted in 0.5% DMSO. Each BDE209 exposure group was supplemented with 1 mM NAC to form the NAC-BDE209 treatment groups, as shown in Table 1.

### 2.5. Cell Morphology Observation

Measurements of the morphological changes in Beas-2B cells exposed to BDE209 using an inverted microscope (Olympus, Tokyo, Japan). At the same time, we need to adjust the brightness, height of the light source, and lens of the instrument. Finally, observe the cells at 40× magnification and capture representative micrographs using the microscope’s integrated camera and software.

### 2.6. Determination of Intracellular GSH, SOD, CAT, MDA

Cells were washed once with PBS, and cell samples were lysed with Western and IP cell lysates (Beyotime Biotechnology, P0013) at a ratio of 100–200 μL of lysate per 1 million cells, followed by centrifugation at 4 °C, 12,000 g for 10 min, and the supernatant was taken for the enzyme activity assay. For specific operating procedures, refer to the kit manual. The assay kits for measuring the activities of GPx (Glutathione Peroxidase Assay Kit), SOD (Superoxide Dismutase Assay Kit), CAT (Catalase Assay Kit), and MDA (Lipid Peroxidation MDA Assay Kit) were purchased from Beyotime Biotechnology in Shanghai, China.

Glutathione Peroxidase (GPx) Assay: GPx activity was measured in 96-well plates according to the manufacturer’s protocol. Briefly, assay buffer, test samples, and GPx detection working solution were sequentially added to the wells and mixed thoroughly. The reaction was initiated by adding 4 µL of 15 mM peroxide substrate solution. After gentle mixing, preferably using a microplate shaker, the absorbance at 340 nm was continuously monitored using a microplate reader (Thermo Scientific, Waltham, MA, USA) at 25 °C.

SOD Activity Assay: The assay was performed in a 96-well plate according to the manufacturer’s instructions. Sample wells and various blank control wells were set up accordingly. Test samples and other required reagents were sequentially added to the designated wells. The reaction was initiated by adding the initiation working solution, followed by thorough mixing. It is important to note that the reaction commences immediately after the addition of the initiation solution. The plate was then incubated for 30 min, and the absorbance was measured at 450 nm using a microplate reader.

CAT Activity Assay: A standard curve was first constructed for the quantification of CAT activity. A series of different concentrations of hydrogen peroxide (H_2_O_2_) standards were prepared by adding varying volumes of the stock solution into a 96-well plate. This was followed by the addition of the chromogenic working solution to each well. The plate was then incubated at 25 °C for a period ranging from 15 to 45 min, after which the absorbance was measured at 520 nm using a microplate reader.

MDA Assay: The MDA content was determined using a commercial assay kit. Briefly, 0.1 mL of PBS was used as the blank control, while 0.1 mL of a series of standard solutions at different concentrations was prepared for generating the standard curve. Subsequently, 0.1 mL of each test sample was aliquoted for measurement. Then, 0.2 mL of the MDA detection working solution was added to each tube according to the manufacturer’s recommended reaction system. After thorough mixing, the mixtures were heated at 100 °C in a boiling water bath for 15 min. Following heating, the tubes were cooled to room temperature in a water bath and centrifuged at 1000× *g* for 10 min at room temperature. Finally, 200 µL of the supernatant from each tube was transferred to a 96-well plate, and the absorbance was measured at 532 nm using a microplate reader.

### 2.7. Determination of Intracellular ROS

Measurements of ROS were performed in the Beas-2B cell using laser confocal microscopy (Carl Zeiss AG, Oberkochen, Germany), the intracellular reactive Oxygen Species Assay Kit (Beyotime, Shanghai, China), and the mitochondrial reactive Oxygen Species Assay Kit (Thermo, Waltham, MA, USA) used to assess ROS levels in Beas-2B cells.

The level of intracellular ROS was measured from the DCFH-DA probe inside the cell, and it detected the fluorescent DCF generated from the oxidation of non-fluorescent DCFH at the excitation wavelength of 488 nm and the emission wavelength of 525 nm. The level of mitochondrial ROS was measured from the oxidation of MitoSOX by superoxide anion in mitochondria to produce red fluorescence at the excitation wavelength of 510 nm and the emission wavelength of 580 nm. After that, an analysis of relative fluorescence intensity was performed using ImageJ software (Version 1.53t) for NIH.

### 2.8. Statistical Analysis

Statistical computations were performed using SPSS software version 26 for IBM. The changes in ROS level and enzyme viability between different treatment groups were compared using one-way ANOVA followed by Waller Duncan’s post hoc test. Fitting of the dose-effect curve and calculation of IC_50_ were generated using RTCA S16. A *p*-value less than 0.05 was considered statistically significant unless otherwise indicated.

## 3. Results and Discussion

### 3.1. Cell Proliferation Toxicity

Cell proliferation is an important indicator for evaluating the cytotoxic effects of pollutants. We plotted the proliferation curves of Beas-2B cells under BDE209 stimulation by using a real-time label-free cell analyzer (RTCA). Electrical impedance measurements of Beas-2B cells were assessed using the normalized cell index (NCI).

**Figure 1:**
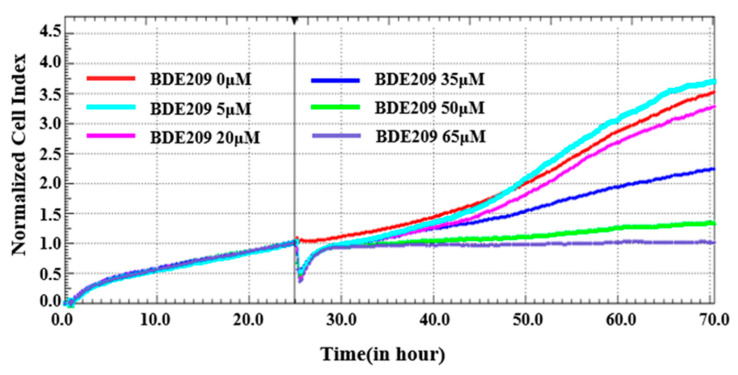
Toxicity of BDE209 on Beas-2B cells detected using RTCA.

As shown in Figure 1, the NCI of normal cells is 3.5. While the concentration of BDE209 reaches 5 µM, a small amount of rise is shown on the NCI of Beas-2B cells. This is the same as the toxic-excitatory effect caused by the previous low concentration and is a stress response by the body to defend itself against the damage caused by the toxicity [21]. While the concentration of BDE209 is enhanced, the NCI of Beas-2B cells displays a dose-dependent decreasing trend. At concentrations up to 35 µM, the NCI decreases to 2.25. At a concentration of 65 µM, the NCI tends to be 1. The results showed that BDE209 inhibits the proliferation of Beas-2B cells. Many studies are in high agreement with us. High levels of exposure damage cells beyond their regulatory levels, leading to increased inhibition of cell proliferation [22,23].

**Figure 2:**
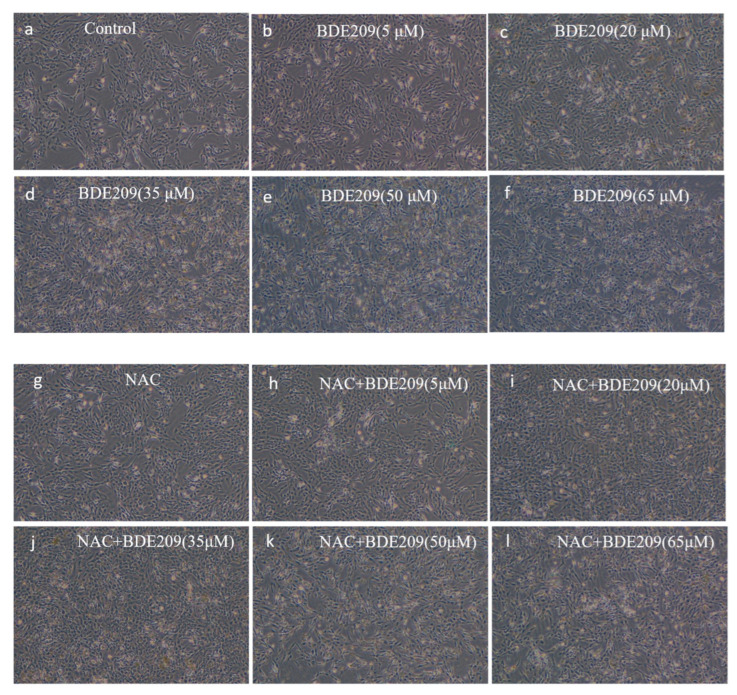
Observed morphological changes in cells stimulated by BDE209 under a 40× inverted microscope ((**a**–**f**): BDE209 exposure group, (**g**–**l**): NAC + BDE209 exposure group).

Morphological observations of cells grown under normal conditions and BDE209 exposure were performed using an inverted microscope. Morphological changes in Beas-2B cells in each group are shown in Figure 2.

Control group cells exhibited good adhesion, normal morphology, and a well-spread, filamentous distribution, with excellent colony growth (Figure 2a). The change in the morphology of Beas-2B cells induced by exposure to low concentrations (<10 µM) of BDE209 does not undergo significant damage (Figure 2b). As the concentration of the BDE209 exposure group increased (>10 µM), cells began to show damage such as edema, deformation, and even apoptosis and shedding. At concentrations of 50 µM and above, cells were almost completely destroyed, with very few adherent cells remaining and virtually no intact cell morphology visible. The degree of damage increases with increasing concentration (Figure 2c–f). Observations showed that BDE209 exposure induces toxic damage in Beas-2B cells, and the damage increases in a dose-dependent manner. We observed that BDE209 exerted some adverse effects on the morphology and proliferation of Beas-2B cells, consistent with a growing body of literature data, including reports of BDE209′s cytotoxicity across various cell types. In the hepatic system, BDE209 induces significant liver injury and inflammation, a process identified as being mediated by ferroptosis [24]. It also promotes growth inhibition and apoptosis in human normal liver L-02 cells [25]. Furthermore, in immune cells such as THP-1 macrophages, BDE209 exposure leads to lipid accumulation and altered cellular morphology and function, while microRNA-21 mitigates these changes by downregulating Toll-like receptor 4 [26].

NAC at 1 mM was added to the BDE209-exposed group at different concentrations. The same increase in the number of Beas-2B cells occurs in the low-concentration NAC + BDE209 group exposure (Figure 2h). The morphology of Beas-2B cells is similarly impaired at high concentrations (Figure 2i–l). On the one hand, NAC did not produce significant changes in cell morphology. This is likely because the primary mechanism of NAC is to modulate intracellular redox status by scavenging reactive oxygen species (ROS) and increasing glutathione levels, rather than directly inducing cytoskeletal or morphological alterations [27]. Its effect is more pronounced on intracellular ROS levels and associated signaling pathways, which may not directly translate to overt changes in cell shape or structure under the present experimental conditions [28]. On the other hand, when the body is damaged beyond its ability to repair itself, even stopping continued stimulation by exogenous pollutants or increasing antioxidants will not restore the body to its original level [29]. The lack of significant changes in cell morphology under NAC addition was because neither the destruction of intracellular biomolecules by the increase in free radicals caused by BDE209 exposure nor the other toxic effects produced are irreversible, and even if the addition of NAC increases the synthesis of reduced GSH, which eliminates some of the toxic effects, it is not able to restore the original morphology of the cells.

### 3.2. ROS Level

Changes in intracellular reactive oxygen species (IROS) and mitochondrial reactive oxygen species (MROS) in normal and damaged cells were observed by using laser confocal microscopy. The blue color represents the nucleus, and the red color represents ROS produced in the cell. ROS were compared by using different fluorescence intensities.

**Figure 3:**
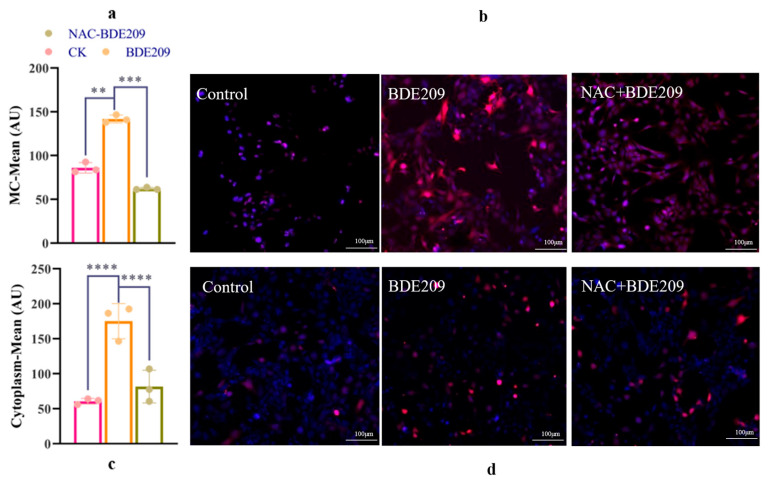
The changes in ROS induced by BDE209 exposure ((**a**,**b**): IROS; (**c**,**d**): MROS). Values represent means ± SD (*n* = 3). Scale bars: 50 µM. ** *p* < 0.01; *** *p* < 0.001, **** *p* < 0.0001.

Figure 3b represents the change in ROS levels in mitochondria. A small amount of red fluorescence was present in the mitochondria of the control group. The red fluorescence for the BDE209 exposure group is more than that for the control group. The red fluorescence for the NAC + BDE209 exposure group is less than that for the BDE209 exposure group. Figure 3d represents the change in ROS levels in the intracellular group. The red fluorescence for the intracellular group is more than that for the mitochondrial group. However, their changes are similar. The red fluorescence of the BDE209-exposed group begins to increase, and the red fluorescence of the NAC + BDE209-exposed group becomes much less than that of the BDE209-exposed group. In summary, BDE209 exposure induced an increase in ROS levels in Beas-2B cells, especially in their mitochondria.

The aim was to perform a quantitative comparison of changes in ROS levels. Whereas mitochondria are the main site of cellular energy conversion and metabolism. From Figure 3a, both control and BED209-exposed Beas-2B cells produce more ROS than that intracytoplasmic. The levels of ROS in mitochondria increase significantly under BDE209 treatment (*p* < 0.0001). The inhibitory effect of NAC on ROS is also very significant (*p* < 0.0001). The levels of ROS for the NAC + BDE209 exposure group are significantly less than that for the BDE209 exposure group (*p* < 0.0001). From Figure 3c, cells normally produce some ROS for intracellular metabolic activities. The levels of ROS are significantly increased in BDE209-exposed cells compared to control Beas-2B cells (*p* < 0.01). The levels of ROS for the NAC + BDE209 exposure group are significantly less than that for the BDE209 exposure group (*p* < 0.001).

The results of the analyses demonstrated that BDE209 exposure induces a large amount of ROS production in Beas-2B cells, leading to oxidative stress. This idea can also be found in other people’s studies [30,31]. Research has found that BDE47-exposed RTG-2 cells have increased ROS levels [32]. The oxidative stress damage associated with elevated ROS levels may have induced apoptosis. Lucio G. C. et al. also found that BDE47 exposure induces ROS in mice and produces oxidative stress [33]. However, the antioxidant NAC significantly reduced the excess ROS produced by mitochondria of Beas-2B cells, indicating that NAC can inhibit the damage of BDE209 to cells. This idea can also be found in the studies of others; Liu et al. investigate the damage induced using rotenone in THP-1 cells. He found that the production of ROS by THP-1 cells pretreated with NAC was reduced [34]. Chen et al. have the same conclusion [35]. The BDE-47-induced increase in ROS levels is inhibited using pretreatment with the ROS scavenger NAC. The quantitative analysis confirmed that BDE209 exposure induces a significant increase in both mitochondrial and cytoplasmic ROS, an effect potently mitigated by the antioxidant NAC. While these findings strongly support the role of oxidative stress in BDE209-induced cytotoxicity, it is crucial to consider the dualistic nature of ROS in cellular metabolism [36,37]. Recent studies have shown that in the advanced stages of cancer, enhancing ROS production can inhibit tumor cell growth and induce apoptosis, which is contrary to the results of this study [37].

### 3.3. Antioxidant Enzyme Activity Levels and Lipid Oxidation Damage

**Figure 4:**
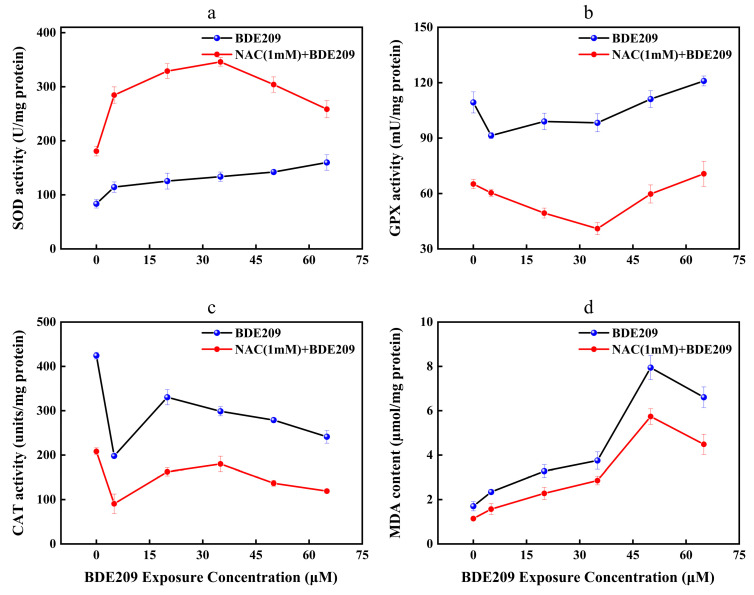
Changes in enzyme activity of Beas-2B cells induced by BDE209 exposure ((**a**): SOD, (**b**): GPX, (**c**): CAT, (**d**): MDA). Values represent means ± SD (*n* = 3).

Antioxidant enzymes play an important role in maintaining redox homeostasis in our body. Analysis of changes in antioxidant-enzyme activity levels is essential. The unpaired electrons of radicals may damage the lipids of cellular membranes, initiated by a process known as lipid peroxidation (LPO) [38]. To testify to the assumption, it was necessary to analyze the changes in the content of MDA. MDA is a product of the lipid peroxidation reaction. NAC as an antioxidant may cause changes in free radical content in Beas-2B cells in vivo. Meanwhile, NAC also affects cellular antioxidant system-related factors [39,40]. Figure 4a–d depicts changes in antioxidant system-related factors in Beas-2B cells exposed to BDE209 at concentrations ranging from 0 to 60 µM, either alone or in combination with 1 mM NAC.

The role of SOD is to disproportionate O^2−^ to H_2_O_2_ [41]. The activities of SOD in the control group are 83.51 U/mg protein. Compared to the control, the levels of SOD after exposure to BDE209 show a concentration-dependent significantly upward trend (Figure 4a). SOD activity is highest at 65 µM with 159.83 U/mg protein. This result conforms with Jian’s research [42]. On the one hand, exposure to pollutants within the body’s tolerance range can induce the activation of the antioxidant-enzyme system [43,44]. On the other hand, the above distinction in SOD may arise from the variation in the unequally biological responses to exogenous pollutants in different organisms and the significant distinction in the sensitivity of SOD to varying levels of oxidative stress [45,46]. After the addition of the antioxidant NAC, the levels of SOD increased to 180.87 U/mg protein. With increasing concentration of BDE209 exposure, the SOD activities increased to 345.93 U/mg protein in 35 µM. However, it took a turn and significantly downregulated to 258.42 U/mg protein. The levels of SOD for the NAC exposure group are still higher than that for the BDE209 exposure group. Mahmoud et al. found that the activities of SOD in the rat brain for the NAC + CPF exposure group are higher than that for the CPF exposure group [47]. Liu et al. also found that NAC treatment can reduce the activities of SOD to attenuate the oxidative damage induced using cold medicine protein (PAT) [48]. The present results demonstrate the effectiveness of NAC in protecting Beas-2B cells against oxidative stress resulting from BDE209 intoxication. This occurred by increasing SOD activity.

Reduced GSH is an important water-soluble antioxidant in animals. GSH can reduce H_2_O_2_ to H_2_O under the action of GPX and oxidize itself to oxidized glutathione (GSSG) [49]. GSSG is catalytically activated by GR and accepts the reduction of H to GSH [50]. The reduction reaction allows the free radical scavenging reaction could be sustained in the body. The analysis of GPX vitality revealed the following findings (Figure 4b). GPX activity exhibits a general trend of inhibition at low doses and activation at high doses. The GPX level in control cells was 109.33 mU/mg protein. The lowest value was observed at a BDE209 concentration of 5 µM (91.38 mU/mg protein). Subsequently, GPX levels began to rise continuously, but remained below control levels at BDE209 concentrations below 35 µM. The maximum value was reached at 65 µM (120.94 mU/mg protein). Our findings differ from those reported by Wang, whose study showed that GPX activity did not exhibit a significant rebound with increasing BDE209 concentrations but remained persistently suppressed [4]. This discrepancy may stem from fundamental differences in the structure and regulatory mechanisms of antioxidant networks between animal and plant cells. The addition of NAC reduced GPX activity. It reached a minimum at 35 μM (40.99/mg protein). Subsequently, GPX activity began to increase, peaking at 65 μM (70.67 mU/mg protein). This may be because when the concentration of BDE209 exceeds 35 μM, the cellular defense system is gradually activated. At this time, excessive ROS are scavenged, and the levels of GPX gradually increase. This result is opposite to Tang’s research, which showed that oxidative stress is due to a combination of different factors and that other factors contribute to enzyme activities [51].

CAT acts similarly to GPX, which catalyzes the dismutation of two molecules of H_2_O_2_ into O_2_ and H_2_O [52]. Compared with the control group (424.32 units/mg protein), CAT activity was significantly inhibited in a concentration-dependent manner by BDE209 exposure compared to the control group (424.32 µM). The most pronounced inhibition is at 5 µM (197.99 units/mg protein), which appears anomalous within the overall dose–response trend. We hypothesize that this may represent a critical threshold where the compound induces significant initial oxidative stress, sufficient to temporarily impair the antioxidant system. When the concentration of BDE209 reached 20 µM, the levels of CAT started to increase (330.36 units/mg protein) and subsequently began to decrease. This implied that the Beas-2B cell following exposure to BDE209 is oxidatively damaged. As shown in Figure 4c, oxidative damage to cells impairs the ability to synthesize and release CAT, leading to a decrease in CAT activities [53]. In addition, the overall change in CAT activities is the result of the antioxidant components scavenging to some extent the oxidative stress induced by exogenous substances. This change is also consistent with the “abnormality-recovery-dysregulation” response pattern of the body when stimulated by exogenous pollutants [54]. The addition of NAC results in a more pronounced inhibition of CAT viabilities (208.16 µM). This result is consistent with Sahar’s research, which showed that NAC fails to attenuate BDE209 exposure-induced oxidative damage by promoting the activities of CAT [47].

MDA can reflect the degree of lipid damage in cells. As shown in Figure 4d, our study found that the amount of MDA in Beas-2B cells under normal conditions is 1.70 µMol/mg protein. The highest content was observed at 50 µM (7.94 µmol/mg protein), with MDA levels increasing significantly in a concentration-dependent manner. This result conforms with Wang’s research [4]. BDE209 induces ROS targeting fatty acids in membrane phospholipids and destroys cellular membranes. Elsewhere it says oxidative damage induced by high concentrations of pollutants tends to overload the scavenging capacity of the enzyme, resulting in a decrease in MDA content. The lack of antioxidant defenses leads to more severe oxidative damage, creating a vicious circle [55]. The addition of NAC resulted in a non-significant decrease in the MDA content of the control group (1.14 µmol/mg protein). In the same situation, as the concentration of BDE209 exposure increases, the MDA content begins to rise, peaking at 50 µM (5.74 µmol/mg protein). Whereas it was still lower than that of the BDE209 group. This suggests that NAC can correct the redox imbalance induced by BDE209 exposure. This result is consistent with Tang’s study, demonstrating that NAC can inhibit ROS production by attenuating BDE209 phosphorylation and reducing enzyme activity [51].

Despite these findings, this study has several limitations that must be acknowledged. First, our research primarily focused on oxidative stress and its direct cytotoxic mechanisms. However, BDE209 may also induce other cellular dysfunctions, such as mitochondrial damage, which could subsequently trigger pro-inflammatory responses; these specific pathways were not directly elucidated in our study. Second, our study did not measure intracellular concentrations of BDE209 or potential biotransformation. Indeed, few studies have explored possible metabolic reactions of BDE209 within cells or the toxic damage caused by its metabolites, representing a significant knowledge gap. Future research should therefore investigate the complex interactions between oxidative stress, mitochondrial dysfunction, and inflammation. Furthermore, elucidating the metabolic pathways of BDE209 and the relative toxicity of its metabolites is essential for comprehensive risk assessment.

## 4. Conclusions

In summary, this study demonstrates that BDE209 induces oxidative stress-mediated cytotoxic effects in Beas-2B cells. The key mechanism involves a significant BDE209-triggered burst of intracellular ROS, which disrupts the antioxidant defense system—evidenced by imbalanced activities of SOD, CAT, and GPX, and elevated lipid peroxidation (MDA). The antioxidant NAC effectively scavenged the excess ROS and partially alleviated the oxidative damage, as indicated by the promoted activities of SOD and GPX and the reduced MDA levels. However, the inability of NAC to fully reverse the cellular damage, coupled with its unexpected suppression of CAT activity, indicates that BDE209 toxicity involves complex pathways beyond ROS scavenging. These findings underscore oxidative stress as a pivotal mechanism in BDE209 toxicity and suggest the involvement of additional, yet to be elucidated, cellular pathways.

## Figures and Tables

**Table 1:** Cell exposure grouping.

	1		2		3		4		5	
	BDE209 (µM)	NAC (mM)	BDE209 (µM)	NAC (mM)	BDE209 (µM)	NAC (mM)	BDE209 (µM)	NAC (mM)	BDE209 (µM)	NAC (mM)
Control	-	-	-	-	-	-	-	-	-	-
BDE209	5	-	20	-	35	-	50	-	65	-
NAC + BDE209	5	1	20	1	35	1	50	1	65	1

Parallel experiments were conducted in each group and the final data were averaged (*n* = 3); The hyphen (-) indicates that nothing was added.

## Data Availability

Data will be made available on request.

## Supplementary Materials

The following supporting information can be downloaded at https://www.mdpi.com/article/10.3390/toxics13110987/s1: Figure S1: The proliferation curve of Beas-2B cells by RTCA under BDE209 stimulation; Figure S2. Time-dependent curve of IC50 (a: 6 h, b: 12 h, c: 24 h, d: 48 h). Table S1: IC50 values of BDE209 at 6, 12, 24, 48 h and the fitting curve R.
