# Supplementary material for: Effects of Decabromodiphenyl Ether (BDE209) Exposure on Toxicity and Oxidative Stress of Beas-2B Cells
Source: Toxics. 2025 Nov 16;13(11):987. doi: 10.3390/toxics13110987 (PMC12656290; doi:10.3390/toxics13110987)
Supplement: Supplementary file 1 [file toxics-13-00987-s001.zip › toxics-3925111-supplementary.pdf]

Supplementary data

Effects of decabromodiphenyl ether (BDE209) exposure on toxicity and oxidative stress of Beas-2B cells

Yanan Zhang[1,2], Ziyu Xiao[2], Pu Mao[3], Fengrui Yang[2], Yingdi Ma[2], Bensen Xian[2], Mingming Fu[2,4,*], Guiying Li[1]

1 Guangdong Basic Research Center of Excellence for Ecological Security and Green Development, Key Laboratory of City Cluster Environmental Safety and Green Development of the Ministry of Education, School of Environmental Science and Engineering, Guangdong University of Technology, Guangzhou 510006, China;

2 Guangxi Key Laboratory of Environmental Pollution Control Theory and Technology, Guilin University of Technology, Guilin, 541006, China

3 State Key Laboratory of Respiratory Disease, Guangzhou Medical University, Guangzhou, 510182, China;

4 University Engineering Research Center of Watershed Protection and Green Development, Guangxi, Guilin University of Technology, Guilin, 541006, China

* E-mail address: mmfu@glut.edu.cn (Mingming Fu)

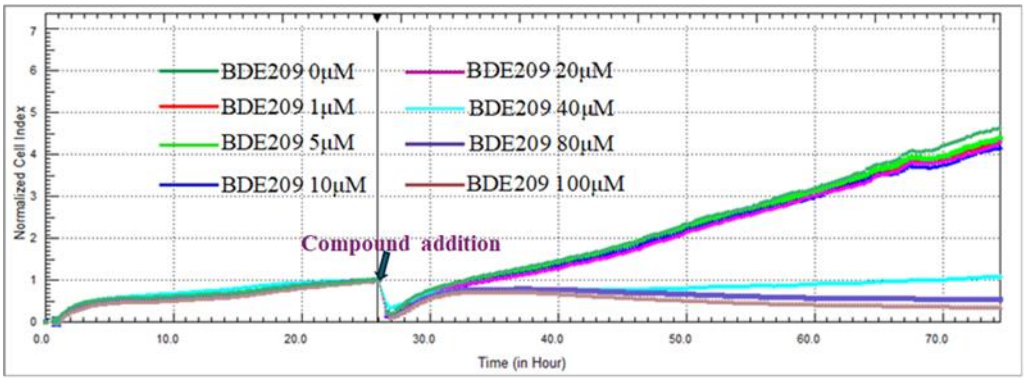

Figure S1. The proliferation curve of Beas-2B cells by RTCA under BDE209 stimulation.

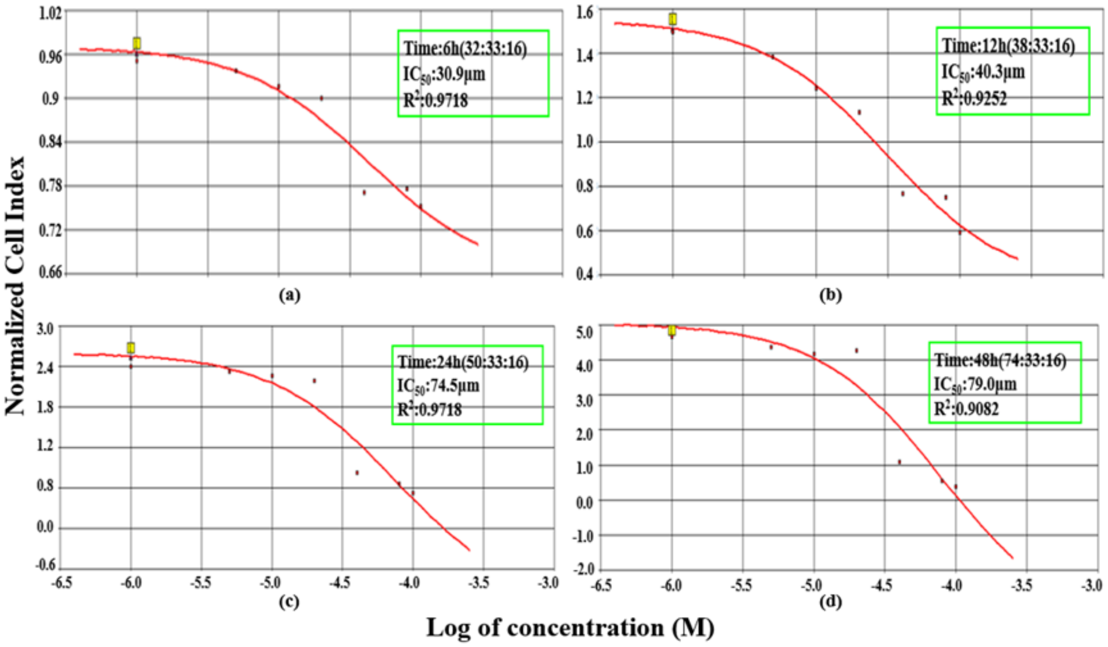

Figure S2. Time-dependent curve of IC50 (a: 6 h, b: 12 h, c: 24 h, d: 48 h).

1.Table S1. IC50 values of BDE209 at 6, 12, 24, 48 h and the fitting curve R.

**Time(h)**	**6**	**12**	**24**	**48**
**IC_{50}(µM)**	30.9	43.0	74.5	79.0
**R^2**	0.9718	0.9252	0.9128	0.9082
